# 2,4,5-Tris(biphenyl-2-yl)-1-bromo­benzene

**DOI:** 10.1107/S1600536811028455

**Published:** 2011-07-23

**Authors:** Ana S. M. C. Rodrigues, Ligia R. Gomes, Luís M. N. B. F. Santos, John Nicolson Low

**Affiliations:** a Centro de Investigação em Química, Departamento de Química e Bioquímica, Faculdade de Ciências, Universidade do Porto, Rua do Campo Alegre, 687, P-4169-007 Porto, Portugal; b REQUIMTE, Departamento de Química e Bioquímica, Faculdade de Ciências, Universidade do Porto, Rua do Campo Alegre, 687, P-4169-007 Porto, Portugal; c Department of Chemistry, University of Aberdeen, Meston Walk, Old Aberdeen AB24 3UE, Scotland

## Abstract

In the title compound, C_42_H_29_Br, the dihedral angles between the central benzene ring and the three attached benzene rings are very similar, lying in the range 52.65 (6)–57.20 (7)°. Of the dihedral angles between the rings of the *o*-biphenyl substituents, two are similar [46.34 (7) and 47.35 (7)°], while the other differs significantly [64.17 (7)°]. In the crystal, mol­ecules are linked into centrosymmetric dimers by two weak C—H⋯π inter­actions.

## Related literature

For background to the Suzuki–Miyaura cross-coupling reaction in the synthesis of aryl­naphthalenes and polyphenyl­enes, see: Miyaura & Suzuki (1995 ▶); Liu *et al.* (2006 ▶); Lima *et al.* (2011 ▶). For crystal structures of related *o*-polyphenyl­enes, see: Muller *et al.* (1997 ▶); Iyer *et al.* (1998 ▶); Nehls *et al.* (2005 ▶).
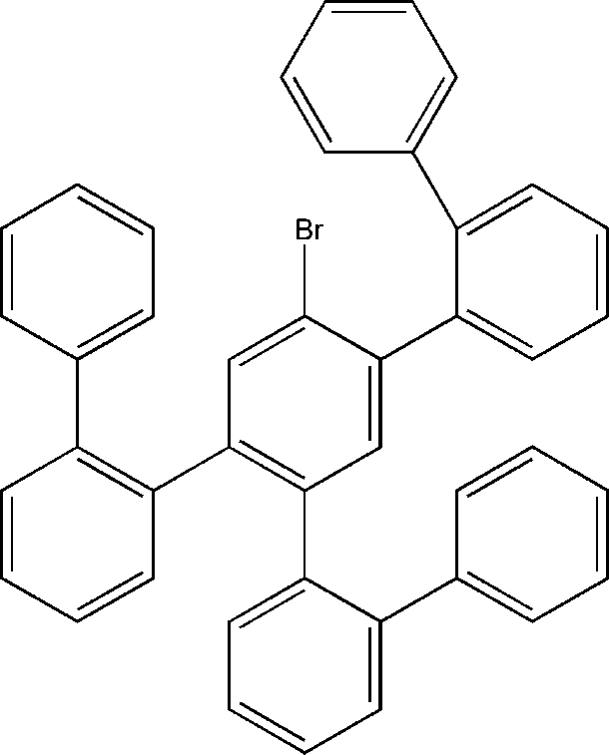


## Experimental

### 

#### Crystal data

C_42_H_29_Br
*M* *_r_* = 613.56
Triclinic, 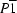

*a* = 11.6723 (5) Å
*b* = 12.2455 (6) Å
*c* = 12.4859 (6) Å
α = 62.549 (2)°
β = 70.771 (2)°
γ = 79.407 (2)°
*V* = 1494.26 (12) Å^3^
*Z* = 2
Mo *K*α radiation
μ = 1.41 mm^−1^
*T* = 150 K
0.30 × 0.20 × 0.10 mm

#### Data collection

Bruker SMART APEX CCD diffractometer
Absorption correction: multi-scan (*SADABS*; Bruker, 2004 ▶) *T*_min_ = 0.678, *T*_max_ = 0.872
37893 measured reflections
8078 independent reflections
7272 reflections with *I* > 2σ(*I*)
*R*_int_ = 0.024

#### Refinement

*R*[*F* ^2^ > 2σ(*F* ^2^)] = 0.027
*wR*(*F* ^2^) = 0.073
*S* = 1.04
8078 reflections
388 parameters
H-atom parameters constrained
Δρ_max_ = 0.45 e Å^−3^
Δρ_min_ = −0.32 e Å^−3^

### 

Data collection: *APEX2* (Bruker, 2004 ▶); cell refinement: *SAINT* (Bruker, 2004 ▶); data reduction: *SAINT*; program(s) used to solve structure: *SHELXS97* (Sheldrick, 2008 ▶); program(s) used to refine structure: *SHELXL97* (Sheldrick, 2008 ▶); molecular graphics: *PLATON* (Spek, 2009) ▶; software used to prepare material for publication: *SHELXL97*.

## Supplementary Material

Crystal structure: contains datablock(s) global, I. DOI: 10.1107/S1600536811028455/hb5952sup1.cif

Structure factors: contains datablock(s) I. DOI: 10.1107/S1600536811028455/hb5952Isup2.hkl

Supplementary material file. DOI: 10.1107/S1600536811028455/hb5952Isup3.cml

Additional supplementary materials:  crystallographic information; 3D view; checkCIF report

## Figures and Tables

**Table 1:** Hydrogen-bond geometry (Å, °) *Cg*2 and *Cg*6 are the centroids of the C21–26 and C421–C426 rings, respectively.

*D*—H⋯*A*	*D*—H	H⋯*A*	*D*⋯*A*	*D*—H⋯*A*
C43—H43⋯*Cg*2^i^	0.95	2.89	3.7273 (15)	148
C226—H226⋯*Cg*6^i^	0.95	2.78	3.6129 (17)	147

Symmetry code: (i) 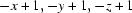.

**Fig. 1.**
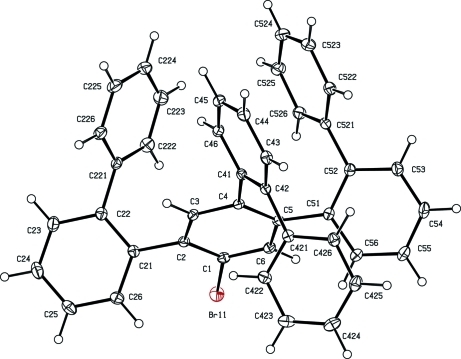
A view of (I) with displacement ellipsoids drawn at the 30% probability level.

**Fig. 2.**
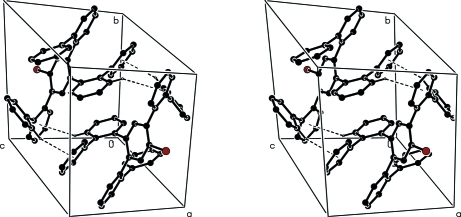
Stereoview of the dimer formed by the pairs of C—H···π interactions. Hydrogen atoms not involved in the motifs are not included.

## supplementary crystallographic information

### Comment

The Suzuki-Miyaura reaction, also called Suzuki coupling, is a particular type of cross-coupling reaction that involves the coupling of an organometallic boron species, R—B, with an organic electrophile, *R*'-*X* where *X* is an organic halide (Br in the presented case), in the presence of a Pd catalyst and a base, resulting in the formation of a new C—C bond, (Miyaura & Suzuki, 1995) and (Liu *et al.*,  2006).

The title compound can thus be used as a useful electrophile for insertion of a new substituent on the 1 position of the central ring and it was prepared as a part of the ongoing research concerning about the importance of the transmetallation step in the overall efficiency of the cross-coupling reaction and about the role of the intramolecular Pd···π and π ··· π aromatic interactions on the stabilization of Pd(II) complexes, that contributes to the selectivity of the Suzuki-Miyaura cross-coupling reaction in the synthesis of arylnaphthalenes and polypnenylenes, (Lima *et al.*,  2011).

The molecule, Fig.1, presents a high degree of torsion between phenyl rings probably due to the steric hindrance promoted by the *ortho*-substitution of the 2-biphenylyl substituents on the central benzene ring. The dihedral angles between the mean plane of the central phenyl ring containing atoms C1 and the mean planes of the phenyl rings attached to atoms C2, C4 and C5 are respectively, 57.20 (7)°, 56.88 (6)° and 52.65 (6)°. The dihedral angle between the phenyl rings containing atoms C21 and C221 is 64.17 (7)°, that between the mean plane of the phenyl rings C containing atoms C41 and C421 is 47.35 (7)°, that between the phenyl rings C containing atoms C51 and C521 is 46.34 (7)° and that between the phenyl rings containing atoms C41 and C51 is 52.21 (7)°.

The molecules are linked to form a centrosymmetric dimer by two weak C—H···π interactions, Table 1 and Fig.2. There are no π···π interactions.

### Experimental

A mixture of 1,2,4,5-tetrabromobenzene (0.35 mmol), 2-boronic acid (1.9 mmol), PdCl_2_(dppe) (0.07 mmol) and K_2_CO_3_ (2.5 mmol) in 35 cm^3^ of distilled water/toluene (1:2.5) was stirred under nitrogen atmosphere for approximately 10 h at 359 K. Afterwards the reaction mixture was extracted twice with aqueous solutions of NaOH (1*M*) followed by HCl (1*M*), respectively. The product was purified by recrystallization from methanol. The sublimation of the compound at reduced pressure (<10 Pa) and T=520 K, produced colorless blocks of the title compound.

### Refinement

H atoms were treated as riding atoms with C—H(aromatic), 0.95 Å, with *U*_iso_ = 1.2Ueq(C). The positions of the H atoms were calculated and checked on a difference map during the refinement. Four refections were omitted, two of which were obscured by the beamstop and two for which (Iobs/Ical)/sigma was greater than 10.

### Crystal data

**Table:**

C_42_H_29_Br	*Z* = 2
*M**_r_* = 613.56	*F*(000) = 632
Triclinic, *P*1	*D*_x_ = 1.364 Mg m^−^^3^
*a* = 11.6723 (5) Å	Mo *K*α radiation, λ = 0.71073 Å
*b* = 12.2455 (6) Å	Cell parameters from 302 reflections
*c* = 12.4859 (6) Å	θ = 3.4–58.4°
α = 62.549 (2)°	µ = 1.41 mm^−^^1^
β = 70.771 (2)°	*T* = 150 K
γ = 79.407 (2)°	Block, colorless
*V* = 1494.26 (12) Å^3^	0.30 × 0.20 × 0.10 mm

### Data collection

**Table:**

Bruker SMART APEX CCD diffractometer	8078 independent reflections
Radiation source: fine-focus sealed tube	7272 reflections with *I* > 2σ(*I*)
graphite	*R*_int_ = 0.024
Detector resolution: 8.33 pixels mm^-1^	θ_max_ = 29.4°, θ_min_ = 2.6°
ω scans	*h* = −16→16
Absorption correction: multi-scan (*SADABS*; Bruker, 2004)	*k* = −16→16
*T*_min_ = 0.678, *T*_max_ = 0.872	*l* = −17→17
37893 measured reflections	

### Refinement

**Table:**

Refinement on *F*^2^	Primary atom site location: structure-invariant direct methods
Least-squares matrix: full	Secondary atom site location: difference Fourier map
*R*[*F*^2^ > 2σ(*F*^2^)] = 0.027	Hydrogen site location: inferred from neighbouring sites
*wR*(*F*^2^) = 0.073	H-atom parameters constrained
*S* = 1.04	*w* = 1/[σ^2^(*F*_o_^2^) + (0.0377*P*)^2^ + 0.5752*P*] where *P* = (*F*_o_^2^ + 2*F*_c_^2^)/3
8078 reflections	(Δ/σ)_max_ = 0.001
388 parameters	Δρ_max_ = 0.45 e Å^−^^3^
0 restraints	Δρ_min_ = −0.32 e Å^−^^3^

### Special details

**Table:**

Geometry. All e.s.d.'s (except the e.s.d. in the dihedral angle between two l.s. planes) are estimated using the full covariance matrix. The cell e.s.d.'s are taken into account individually in the estimation of e.s.d.'s in distances, angles and torsion angles; correlations between e.s.d.'s in cell parameters are only used when they are defined by crystal symmetry. An approximate (isotropic) treatment of cell e.s.d.'s is used for estimating e.s.d.'s involving l.s. planes.
Refinement. Refinement of *F*^2^ against ALL reflections. The weighted *R*-factor *wR* and goodness of fit *S* are based on *F*^2^, conventional *R*-factors *R* are based on *F*, with *F* set to zero for negative *F*^2^. The threshold expression of *F*^2^ > σ(*F*^2^) is used only for calculating *R*-factors(gt) *etc*. and is not relevant to the choice of reflections for refinement. *R*-factors based on *F*^2^ are statistically about twice as large as those based on *F*, and *R*- factors based on ALL data will be even larger.

### Fractional atomic coordinates and isotropic or equivalent isotropic displacement parameters (Å^2^)

**Table:**

	*x*	*y*	*z*	*U*_iso_*/*U*_eq_	
C1	0.04428 (10)	0.52859 (11)	0.66271 (11)	0.0180 (2)	
Br11	−0.115818 (11)	0.483970 (12)	0.695774 (14)	0.02699 (5)	
C2	0.13584 (11)	0.43876 (11)	0.69266 (11)	0.0170 (2)	
C21	0.12150 (10)	0.30322 (11)	0.74820 (12)	0.0179 (2)	
C22	0.15497 (11)	0.22407 (11)	0.85852 (12)	0.0200 (2)	
C221	0.19140 (11)	0.27166 (11)	0.93255 (11)	0.0196 (2)	
C222	0.10917 (12)	0.33974 (13)	0.99043 (13)	0.0266 (3)	
H222	0.0293	0.3574	0.9813	0.032*	
C223	0.14269 (15)	0.38203 (14)	1.06127 (14)	0.0320 (3)	
H223	0.0853	0.4269	1.1017	0.038*	
C224	0.25946 (15)	0.35897 (14)	1.07310 (14)	0.0323 (3)	
H224	0.2827	0.3886	1.1208	0.039*	
C225	0.34230 (14)	0.29245 (14)	1.01508 (14)	0.0311 (3)	
H225	0.4229	0.2773	1.0222	0.037*	
C226	0.30824 (12)	0.24769 (13)	0.94644 (13)	0.0254 (3)	
H226	0.3651	0.2004	0.9087	0.030*	
C23	0.15294 (13)	0.09674 (12)	0.90096 (14)	0.0284 (3)	
H23	0.1758	0.0428	0.9753	0.034*	
C24	0.11824 (14)	0.04787 (13)	0.83645 (16)	0.0321 (3)	
H24	0.1180	−0.0389	0.8662	0.038*	
C25	0.08388 (13)	0.12577 (13)	0.72852 (15)	0.0282 (3)	
H25	0.0593	0.0926	0.6845	0.034*	
C26	0.08542 (12)	0.25264 (12)	0.68466 (13)	0.0231 (2)	
H26	0.0617	0.3057	0.6106	0.028*	
C3	0.24967 (10)	0.48201 (11)	0.66557 (11)	0.0167 (2)	
H3	0.3141	0.4232	0.6846	0.020*	
C4	0.27401 (10)	0.60642 (11)	0.61227 (11)	0.0157 (2)	
C41	0.40053 (10)	0.63552 (10)	0.59165 (11)	0.0165 (2)	
C42	0.47576 (11)	0.71026 (11)	0.47357 (11)	0.0172 (2)	
C421	0.43770 (11)	0.76334 (11)	0.35645 (11)	0.0181 (2)	
C422	0.38465 (12)	0.69133 (12)	0.32675 (12)	0.0228 (2)	
H422	0.3721	0.6067	0.3823	0.027*	
C423	0.35023 (13)	0.74304 (14)	0.21627 (13)	0.0287 (3)	
H423	0.3140	0.6935	0.1969	0.034*	
C424	0.36832 (13)	0.86627 (15)	0.13420 (13)	0.0299 (3)	
H424	0.3439	0.9014	0.0592	0.036*	
C425	0.42225 (13)	0.93801 (13)	0.16230 (13)	0.0275 (3)	
H425	0.4351	1.0225	0.1062	0.033*	
C426	0.45744 (12)	0.88690 (12)	0.27201 (12)	0.0224 (2)	
H426	0.4954	0.9364	0.2899	0.027*	
C43	0.59232 (11)	0.73265 (11)	0.46560 (13)	0.0214 (2)	
H43	0.6428	0.7850	0.3868	0.026*	
C44	0.63552 (12)	0.67984 (12)	0.57061 (14)	0.0247 (3)	
H44	0.7150	0.6959	0.5633	0.030*	
C45	0.56246 (12)	0.60375 (12)	0.68599 (13)	0.0238 (3)	
H45	0.5920	0.5665	0.7579	0.029*	
C46	0.44577 (11)	0.58210 (11)	0.69626 (12)	0.0201 (2)	
H46	0.3959	0.5301	0.7757	0.024*	
C5	0.17909 (11)	0.69510 (11)	0.58414 (11)	0.0163 (2)	
C51	0.19295 (11)	0.83131 (11)	0.52144 (11)	0.0178 (2)	
C52	0.24172 (11)	0.89620 (11)	0.56378 (11)	0.0190 (2)	
C521	0.27742 (11)	0.83805 (11)	0.68262 (12)	0.0191 (2)	
C522	0.38617 (12)	0.86815 (12)	0.68500 (13)	0.0237 (3)	
H522	0.4379	0.9240	0.6096	0.028*	
C523	0.41975 (14)	0.81785 (13)	0.79547 (14)	0.0288 (3)	
H523	0.4945	0.8384	0.7952	0.035*	
C524	0.34422 (15)	0.73745 (14)	0.90642 (14)	0.0303 (3)	
H524	0.3670	0.7028	0.9823	0.036*	
C525	0.23521 (14)	0.70788 (13)	0.90606 (13)	0.0277 (3)	
H525	0.1830	0.6534	0.9821	0.033*	
C526	0.20186 (12)	0.75741 (12)	0.79510 (12)	0.0226 (2)	
H526	0.1272	0.7363	0.7958	0.027*	
C53	0.25388 (13)	1.02375 (12)	0.49236 (13)	0.0243 (3)	
H53	0.2891	1.0677	0.5188	0.029*	
C54	0.21601 (14)	1.08736 (12)	0.38426 (13)	0.0278 (3)	
H54	0.2251	1.1738	0.3377	0.033*	
C55	0.16479 (13)	1.02411 (13)	0.34442 (13)	0.0271 (3)	
H55	0.1371	1.0671	0.2714	0.033*	
C56	0.15443 (12)	0.89755 (12)	0.41216 (12)	0.0220 (2)	
H56	0.1203	0.8545	0.3838	0.026*	
C6	0.06467 (11)	0.65329 (11)	0.61030 (11)	0.0185 (2)	
H6	−0.0003	0.7115	0.5918	0.022*	

### Atomic displacement parameters (Å^2^)

**Table:**

	*U*^11^	*U*^22^	*U*^33^	*U*^12^	*U*^13^	*U*^23^
C1	0.0140 (5)	0.0221 (6)	0.0188 (6)	−0.0031 (4)	−0.0054 (4)	−0.0081 (5)
Br11	0.01578 (6)	0.02769 (7)	0.03709 (9)	−0.00372 (5)	−0.00922 (5)	−0.01137 (6)
C2	0.0183 (5)	0.0169 (5)	0.0154 (5)	−0.0029 (4)	−0.0051 (4)	−0.0057 (4)
C21	0.0149 (5)	0.0178 (5)	0.0202 (6)	−0.0028 (4)	−0.0035 (4)	−0.0077 (5)
C22	0.0181 (5)	0.0186 (6)	0.0215 (6)	−0.0013 (4)	−0.0051 (4)	−0.0073 (5)
C221	0.0226 (6)	0.0162 (5)	0.0164 (5)	−0.0011 (4)	−0.0063 (4)	−0.0033 (4)
C222	0.0233 (6)	0.0290 (7)	0.0254 (7)	0.0011 (5)	−0.0054 (5)	−0.0118 (6)
C223	0.0408 (8)	0.0300 (7)	0.0255 (7)	0.0021 (6)	−0.0071 (6)	−0.0149 (6)
C224	0.0488 (9)	0.0271 (7)	0.0243 (7)	−0.0052 (6)	−0.0167 (6)	−0.0079 (6)
C225	0.0326 (7)	0.0319 (7)	0.0304 (7)	0.0005 (6)	−0.0185 (6)	−0.0090 (6)
C226	0.0250 (6)	0.0260 (6)	0.0249 (7)	0.0048 (5)	−0.0114 (5)	−0.0100 (5)
C23	0.0323 (7)	0.0186 (6)	0.0311 (7)	0.0005 (5)	−0.0122 (6)	−0.0064 (5)
C24	0.0344 (7)	0.0191 (6)	0.0434 (9)	−0.0018 (5)	−0.0110 (6)	−0.0137 (6)
C25	0.0248 (6)	0.0285 (7)	0.0391 (8)	−0.0039 (5)	−0.0076 (6)	−0.0208 (6)
C26	0.0206 (6)	0.0255 (6)	0.0259 (6)	−0.0031 (5)	−0.0073 (5)	−0.0120 (5)
C3	0.0161 (5)	0.0167 (5)	0.0164 (5)	−0.0003 (4)	−0.0060 (4)	−0.0056 (4)
C4	0.0156 (5)	0.0171 (5)	0.0142 (5)	−0.0019 (4)	−0.0050 (4)	−0.0057 (4)
C41	0.0155 (5)	0.0143 (5)	0.0208 (6)	−0.0006 (4)	−0.0065 (4)	−0.0076 (4)
C42	0.0164 (5)	0.0156 (5)	0.0206 (6)	−0.0006 (4)	−0.0053 (4)	−0.0086 (4)
C421	0.0159 (5)	0.0188 (5)	0.0175 (5)	−0.0021 (4)	−0.0018 (4)	−0.0077 (5)
C422	0.0244 (6)	0.0237 (6)	0.0199 (6)	−0.0065 (5)	−0.0026 (5)	−0.0094 (5)
C423	0.0278 (7)	0.0389 (8)	0.0229 (6)	−0.0098 (6)	−0.0053 (5)	−0.0144 (6)
C424	0.0271 (7)	0.0396 (8)	0.0190 (6)	−0.0005 (6)	−0.0070 (5)	−0.0094 (6)
C425	0.0302 (7)	0.0230 (6)	0.0201 (6)	0.0000 (5)	−0.0040 (5)	−0.0041 (5)
C426	0.0234 (6)	0.0194 (6)	0.0219 (6)	−0.0031 (5)	−0.0032 (5)	−0.0082 (5)
C43	0.0167 (5)	0.0191 (6)	0.0271 (6)	−0.0029 (4)	−0.0039 (5)	−0.0097 (5)
C44	0.0176 (6)	0.0234 (6)	0.0376 (7)	−0.0004 (5)	−0.0121 (5)	−0.0141 (6)
C45	0.0240 (6)	0.0214 (6)	0.0304 (7)	0.0025 (5)	−0.0166 (5)	−0.0100 (5)
C46	0.0216 (6)	0.0168 (5)	0.0211 (6)	−0.0007 (4)	−0.0091 (5)	−0.0054 (5)
C5	0.0176 (5)	0.0166 (5)	0.0149 (5)	−0.0007 (4)	−0.0061 (4)	−0.0059 (4)
C51	0.0165 (5)	0.0163 (5)	0.0181 (5)	0.0003 (4)	−0.0049 (4)	−0.0057 (4)
C52	0.0197 (5)	0.0169 (5)	0.0182 (6)	−0.0006 (4)	−0.0044 (4)	−0.0064 (5)
C521	0.0241 (6)	0.0158 (5)	0.0191 (6)	−0.0014 (4)	−0.0065 (5)	−0.0084 (5)
C522	0.0274 (6)	0.0194 (6)	0.0242 (6)	−0.0057 (5)	−0.0076 (5)	−0.0075 (5)
C523	0.0325 (7)	0.0275 (7)	0.0325 (7)	−0.0055 (5)	−0.0151 (6)	−0.0122 (6)
C524	0.0410 (8)	0.0294 (7)	0.0245 (7)	−0.0038 (6)	−0.0160 (6)	−0.0095 (6)
C525	0.0342 (7)	0.0273 (7)	0.0187 (6)	−0.0072 (5)	−0.0060 (5)	−0.0064 (5)
C526	0.0254 (6)	0.0221 (6)	0.0199 (6)	−0.0048 (5)	−0.0053 (5)	−0.0081 (5)
C53	0.0294 (6)	0.0174 (6)	0.0238 (6)	−0.0026 (5)	−0.0059 (5)	−0.0075 (5)
C54	0.0342 (7)	0.0158 (6)	0.0245 (7)	0.0008 (5)	−0.0068 (5)	−0.0030 (5)
C55	0.0301 (7)	0.0231 (6)	0.0220 (6)	0.0040 (5)	−0.0108 (5)	−0.0041 (5)
C56	0.0223 (6)	0.0217 (6)	0.0212 (6)	0.0018 (5)	−0.0097 (5)	−0.0071 (5)
C6	0.0167 (5)	0.0199 (6)	0.0191 (6)	0.0012 (4)	−0.0073 (4)	−0.0078 (5)

### Geometric parameters (Å, °)

**Table:**

C1—C6	1.3879 (17)	C423—C424	1.386 (2)
C1—C2	1.3943 (17)	C423—H423	0.9500
C1—Br11	1.9027 (11)	C424—C425	1.387 (2)
C2—C3	1.3993 (16)	C424—H424	0.9500
C2—C21	1.4927 (16)	C425—C426	1.3867 (19)
C21—C26	1.3998 (17)	C425—H425	0.9500
C21—C22	1.4049 (17)	C426—H426	0.9500
C22—C23	1.3986 (18)	C43—C44	1.3875 (19)
C22—C221	1.4903 (18)	C43—H43	0.9500
C221—C226	1.3921 (18)	C44—C45	1.384 (2)
C221—C222	1.3939 (18)	C44—H44	0.9500
C222—C223	1.389 (2)	C45—C46	1.3885 (17)
C222—H222	0.9500	C45—H45	0.9500
C223—C224	1.383 (2)	C46—H46	0.9500
C223—H223	0.9500	C5—C6	1.4008 (16)
C224—C225	1.384 (2)	C5—C51	1.4944 (16)
C224—H224	0.9500	C51—C56	1.4041 (17)
C225—C226	1.390 (2)	C51—C52	1.4095 (17)
C225—H225	0.9500	C52—C53	1.4032 (17)
C226—H226	0.9500	C52—C521	1.4854 (17)
C23—C24	1.385 (2)	C521—C526	1.3968 (17)
C23—H23	0.9500	C521—C522	1.3982 (18)
C24—C25	1.385 (2)	C522—C523	1.3855 (19)
C24—H24	0.9500	C522—H522	0.9500
C25—C26	1.3904 (19)	C523—C524	1.387 (2)
C25—H25	0.9500	C523—H523	0.9500
C26—H26	0.9500	C524—C525	1.387 (2)
C3—C4	1.3944 (16)	C524—H524	0.9500
C3—H3	0.9500	C525—C526	1.3902 (19)
C4—C5	1.4115 (16)	C525—H525	0.9500
C4—C41	1.4943 (15)	C526—H526	0.9500
C41—C46	1.3973 (17)	C53—C54	1.386 (2)
C41—C42	1.4075 (17)	C53—H53	0.9500
C42—C43	1.3996 (16)	C54—C55	1.388 (2)
C42—C421	1.4845 (17)	C54—H54	0.9500
C421—C426	1.3969 (17)	C55—C56	1.3874 (19)
C421—C422	1.3971 (17)	C55—H55	0.9500
C422—C423	1.3897 (19)	C56—H56	0.9500
C422—H422	0.9500	C6—H6	0.9500
			
C6—C1—C2	121.96 (11)	C423—C424—C425	119.58 (13)
C6—C1—Br11	117.21 (9)	C423—C424—H424	120.2
C2—C1—Br11	120.83 (9)	C425—C424—H424	120.2
C1—C2—C3	115.95 (11)	C426—C425—C424	120.23 (13)
C1—C2—C21	125.21 (11)	C426—C425—H425	119.9
C3—C2—C21	118.84 (10)	C424—C425—H425	119.9
C26—C21—C22	119.17 (11)	C425—C426—C421	120.66 (12)
C26—C21—C2	120.24 (11)	C425—C426—H426	119.7
C22—C21—C2	120.32 (11)	C421—C426—H426	119.7
C23—C22—C21	119.07 (12)	C44—C43—C42	121.28 (12)
C23—C22—C221	118.93 (12)	C44—C43—H43	119.4
C21—C22—C221	122.00 (11)	C42—C43—H43	119.4
C226—C221—C222	118.60 (12)	C45—C44—C43	119.81 (12)
C226—C221—C22	120.51 (11)	C45—C44—H44	120.1
C222—C221—C22	120.89 (12)	C43—C44—H44	120.1
C223—C222—C221	120.76 (13)	C44—C45—C46	119.78 (12)
C223—C222—H222	119.6	C44—C45—H45	120.1
C221—C222—H222	119.6	C46—C45—H45	120.1
C224—C223—C222	120.10 (14)	C45—C46—C41	121.10 (12)
C224—C223—H223	119.9	C45—C46—H46	119.5
C222—C223—H223	119.9	C41—C46—H46	119.5
C223—C224—C225	119.67 (14)	C6—C5—C4	118.00 (11)
C223—C224—H224	120.2	C6—C5—C51	117.08 (10)
C225—C224—H224	120.2	C4—C5—C51	124.82 (10)
C224—C225—C226	120.34 (14)	C56—C51—C52	118.68 (11)
C224—C225—H225	119.8	C56—C51—C5	116.49 (11)
C226—C225—H225	119.8	C52—C51—C5	124.83 (11)
C225—C226—C221	120.49 (13)	C53—C52—C51	118.58 (12)
C225—C226—H226	119.8	C53—C52—C521	117.64 (11)
C221—C226—H226	119.8	C51—C52—C521	123.74 (11)
C24—C23—C22	121.17 (13)	C526—C521—C522	118.38 (12)
C24—C23—H23	119.4	C526—C521—C52	121.52 (11)
C22—C23—H23	119.4	C522—C521—C52	120.03 (11)
C25—C24—C23	119.82 (13)	C523—C522—C521	121.11 (12)
C25—C24—H24	120.1	C523—C522—H522	119.4
C23—C24—H24	120.1	C521—C522—H522	119.4
C24—C25—C26	119.90 (13)	C522—C523—C524	119.96 (13)
C24—C25—H25	120.0	C522—C523—H523	120.0
C26—C25—H25	120.0	C524—C523—H523	120.0
C25—C26—C21	120.86 (13)	C523—C524—C525	119.68 (13)
C25—C26—H26	119.6	C523—C524—H524	120.2
C21—C26—H26	119.6	C525—C524—H524	120.2
C4—C3—C2	123.83 (11)	C524—C525—C526	120.44 (13)
C4—C3—H3	118.1	C524—C525—H525	119.8
C2—C3—H3	118.1	C526—C525—H525	119.8
C3—C4—C5	118.86 (10)	C525—C526—C521	120.42 (12)
C3—C4—C41	116.45 (10)	C525—C526—H526	119.8
C5—C4—C41	124.68 (10)	C521—C526—H526	119.8
C46—C41—C42	119.23 (11)	C54—C53—C52	121.82 (13)
C46—C41—C4	117.07 (11)	C54—C53—H53	119.1
C42—C41—C4	123.68 (11)	C52—C53—H53	119.1
C43—C42—C41	118.76 (11)	C53—C54—C55	119.64 (12)
C43—C42—C421	118.64 (11)	C53—C54—H54	120.2
C41—C42—C421	122.58 (10)	C55—C54—H54	120.2
C426—C421—C422	118.75 (12)	C56—C55—C54	119.43 (13)
C426—C421—C42	119.91 (11)	C56—C55—H55	120.3
C422—C421—C42	121.32 (11)	C54—C55—H55	120.3
C423—C422—C421	120.24 (12)	C55—C56—C51	121.80 (12)
C423—C422—H422	119.9	C55—C56—H56	119.1
C421—C422—H422	119.9	C51—C56—H56	119.1
C424—C423—C422	120.52 (13)	C1—C6—C5	121.40 (11)
C424—C423—H423	119.7	C1—C6—H6	119.3
C422—C423—H423	119.7	C5—C6—H6	119.3
			
C6—C1—C2—C3	−0.59 (18)	C421—C422—C423—C424	0.2 (2)
Br11—C1—C2—C3	−179.28 (9)	C422—C423—C424—C425	0.6 (2)
C6—C1—C2—C21	−179.52 (11)	C423—C424—C425—C426	−0.2 (2)
Br11—C1—C2—C21	1.79 (17)	C424—C425—C426—C421	−0.9 (2)
C1—C2—C21—C26	59.25 (17)	C422—C421—C426—C425	1.63 (19)
C3—C2—C21—C26	−119.64 (13)	C42—C421—C426—C425	−179.87 (12)
C1—C2—C21—C22	−126.75 (13)	C41—C42—C43—C44	1.91 (18)
C3—C2—C21—C22	54.35 (16)	C421—C42—C43—C44	−176.32 (11)
C26—C21—C22—C23	0.88 (18)	C42—C43—C44—C45	−0.23 (19)
C2—C21—C22—C23	−173.18 (12)	C43—C44—C45—C46	−0.9 (2)
C26—C21—C22—C221	−178.48 (11)	C44—C45—C46—C41	0.28 (19)
C2—C21—C22—C221	7.46 (18)	C42—C41—C46—C45	1.41 (18)
C23—C22—C221—C226	63.90 (17)	C4—C41—C46—C45	179.89 (11)
C21—C22—C221—C226	−116.74 (14)	C3—C4—C5—C6	−0.61 (17)
C23—C22—C221—C222	−115.38 (15)	C41—C4—C5—C6	−179.22 (11)
C21—C22—C221—C222	63.98 (17)	C3—C4—C5—C51	−176.91 (11)
C226—C221—C222—C223	−0.5 (2)	C41—C4—C5—C51	4.48 (19)
C22—C221—C222—C223	178.81 (12)	C6—C5—C51—C56	−51.39 (15)
C221—C222—C223—C224	1.3 (2)	C4—C5—C51—C56	124.94 (13)
C222—C223—C224—C225	−0.7 (2)	C6—C5—C51—C52	128.89 (13)
C223—C224—C225—C226	−0.7 (2)	C4—C5—C51—C52	−54.77 (18)
C224—C225—C226—C221	1.6 (2)	C56—C51—C52—C53	−2.32 (18)
C222—C221—C226—C225	−0.9 (2)	C5—C51—C52—C53	177.39 (12)
C22—C221—C226—C225	179.77 (12)	C56—C51—C52—C521	175.50 (11)
C21—C22—C23—C24	−0.2 (2)	C5—C51—C52—C521	−4.79 (19)
C221—C22—C23—C24	179.16 (13)	C53—C52—C521—C526	131.55 (13)
C22—C23—C24—C25	−0.5 (2)	C51—C52—C521—C526	−46.28 (18)
C23—C24—C25—C26	0.6 (2)	C53—C52—C521—C522	−45.34 (17)
C24—C25—C26—C21	0.1 (2)	C51—C52—C521—C522	136.82 (13)
C22—C21—C26—C25	−0.81 (19)	C526—C521—C522—C523	1.13 (19)
C2—C21—C26—C25	173.25 (12)	C52—C521—C522—C523	178.12 (12)
C1—C2—C3—C4	0.18 (18)	C521—C522—C523—C524	−0.9 (2)
C21—C2—C3—C4	179.18 (11)	C522—C523—C524—C525	0.0 (2)
C2—C3—C4—C5	0.42 (18)	C523—C524—C525—C526	0.6 (2)
C2—C3—C4—C41	179.14 (11)	C524—C525—C526—C521	−0.3 (2)
C3—C4—C41—C46	−55.82 (15)	C522—C521—C526—C525	−0.56 (19)
C5—C4—C41—C46	122.82 (13)	C52—C521—C526—C525	−177.50 (12)
C3—C4—C41—C42	122.58 (12)	C51—C52—C53—C54	2.0 (2)
C5—C4—C41—C42	−58.78 (17)	C521—C52—C53—C54	−175.97 (12)
C46—C41—C42—C43	−2.47 (17)	C52—C53—C54—C55	−0.2 (2)
C4—C41—C42—C43	179.17 (11)	C53—C54—C55—C56	−1.3 (2)
C46—C41—C42—C421	175.69 (11)	C54—C55—C56—C51	0.9 (2)
C4—C41—C42—C421	−2.68 (18)	C52—C51—C56—C55	0.95 (19)
C43—C42—C421—C426	−47.34 (16)	C5—C51—C56—C55	−178.78 (12)
C41—C42—C421—C426	134.51 (12)	C2—C1—C6—C5	0.40 (19)
C43—C42—C421—C422	131.12 (13)	Br11—C1—C6—C5	179.14 (9)
C41—C42—C421—C422	−47.03 (17)	C4—C5—C6—C1	0.22 (18)
C426—C421—C422—C423	−1.26 (19)	C51—C5—C6—C1	176.82 (11)
C42—C421—C422—C423	−179.74 (12)		

### Hydrogen-bond geometry (Å, °)

**Table:**

Cg2 and Cg6 are the centroids of the C21–26 and C421–C426 rings, respectively.

**Table:**

*D*—H···*A*	*D*—H	H···*A*	*D*···*A*	*D*—H···*A*
C43—H43···Cg2^i^	0.95	2.89	3.7273 (15)	148
C226—H226···Cg6^i^	0.95	2.78	3.6129 (17)	147

Symmetry codes: (i) −*x*+1, −*y*+1, −*z*+1.
